# Adjunctive Acupuncture and Moxibustion for Pain Control Due to Recurrent Posterior Shoulder Dislocation in a Female Soccer Player: A Case Report

**DOI:** 10.7759/cureus.105471

**Published:** 2026-03-18

**Authors:** Keisaku Kimura, Yusuke Murakoshi, Daichi Kasuya

**Affiliations:** 1 Acupuncture and Moxibustion, Niigata University of Health and Welfare, Niigata, JPN

**Keywords:** acupuncture and moxbustion, conditioning, female soccer player, pressure pain threshold, recurrent shoulder dislocation

## Abstract

Recurrent shoulder dislocation occurs in soccer when the shoulder joint is subjected to external forces during falls or contact with opposing players. We administered acupuncture treatment to a soccer player complaining of pain as a sequela of dislocation. The results showed that shoulder pain decreased, allowing the player to perform at their usual level. This suggests that acupuncture treatment, when combined with other manual therapies and inner muscle training, may be effective for conditioning.

## Introduction

The shoulder joint is the most susceptible to dislocation of all joints and is among the most frequently encountered sports injuries in track and field. Recurrent shoulder dislocation (RSD) refers to a condition where dislocation occurs two or more times, including the initial dislocation, or where dislocation repeatedly occurs with minimal force. Anterior dislocation accounts for over 95% of cases, while posterior dislocation constitutes 2% to 4% [1,2]. Most cases involve instability resulting from traumatic disruption of shoulder joint stability. Traumatic instability frequently occurs in athletes in contact sports such as rugby and combat sports, and recurrent dislocations can adversely affect an athlete's competitive career [3,4]. Therefore, rehabilitation aimed at improving instability through strengthening muscles and enhancing flexibility is critically important.

Regarding musculoskeletal pain management via acupuncture and moxibustion treatment, a systematic review of clinical practice guidelines supports acupuncture, particularly for chronic musculoskeletal shoulder pain [5]. Furthermore, for impingement syndrome and rotator cuff disorders causing chronic shoulder pain, acupuncture has been reported to provide pain relief and improve shoulder function and disability [6,7]. Its analgesic mechanisms are explained through local reactions, modulation of nociception in the spinal cord and central nervous system, and regulation of endogenous opioids and brain networks [8]. This case study aims to determine whether acupuncture treatment combined with rehabilitation is useful for conditioning athletes experiencing shoulder pain as a sequela of RSD.

Our patient is a female soccer player whose rehabilitation alone showed no effect, but improvement was achieved when acupuncture treatment was combined with rehabilitation. This suggests that acupuncture may have reduced discomfort during movement by relaxing the scapular stabilizing muscles and dynamic stabilizers through pain modulation, rather than treating the structural instability itself. Therefore, acupuncture treatment is considered a useful adjunctive approach to ongoing rehabilitation support for athletes with shoulder pain due to sequelae of RSD.

## Case presentation

A 21-year-old female soccer player presented to the university acupuncture and moxibustion treatment center with shoulder pain due to sequelae of recurrent posterior shoulder dislocation. She was 164 cm tall and weighed 55 kg. Her position was side defender, and her soccer schedule consisted of two hours of practice daily and matches on Saturdays and Sundays, with Mondays as a rest day. She was not on any medication. At age 14, she collided with an opposing player and fell. During this fall, her left shoulder landed in an adducted and internally rotated position. She visited a nearby orthopedic clinic, where a diagnosis of left posterior shoulder dislocation was made, and manual reduction was performed. At age 17, she dislocated the same shoulder again and reduced it herself.

Recently, she developed pain during kicking and heading motions while playing soccer, when sleeping on the left side, and when turning over in bed. The main symptom was worsening pain when turning over while sleeping on the left side. Pain worsened when extending and internally rotating the left shoulder joint from a flexed position. She underwent a left unstable test and a left rotator cuff stress test. Apart from a history of recurrent shoulder stiffness, the patient's shoulder joint range of motion (flexion, extension, adduction, abduction, internal rotation, and external rotation) was within normal limits bilaterally, with no evidence of joint laxity. The patient also had a history of injury at age 16 involving a right lateral malleolus avulsion fracture and a right third metatarsal stress fracture, both of which are now fully healed and caused no current problems.

The patient underwent rehabilitation with a sports trainer; however, the pain did not improve. At the patient's request, acupuncture treatment was initiated for pain relief while the existing rehabilitation program continued. Table 1 describes the timeline from the onset of shoulder dislocation to the initiation of acupuncture treatment. 

**Table 1:** Timeline of left shoulder injury history and treatment

Period	Event
Initial injury	At age 14, the patient fell on her left shoulder and dislocated the shoulder joint while playing soccer.
After injury	(1) Received reduction treatment at the orthopedic department
	(2) Started rehabilitation (strengthening and range of motion exercises for the shoulder joint muscles)
Repeat injury	At age 17, dislocated left shoulder again while playing soccer; self-reduced dislocation
Initiation of acupuncture and moxibustion therapy	Combined this therapy with ongoing rehabilitation to reduce pain
Duration of acupuncture and moxibustion therapy	A total of eight treatments at seven to 10-day intervals

Acupuncture and moxibustion treatment

**Figure 1:**
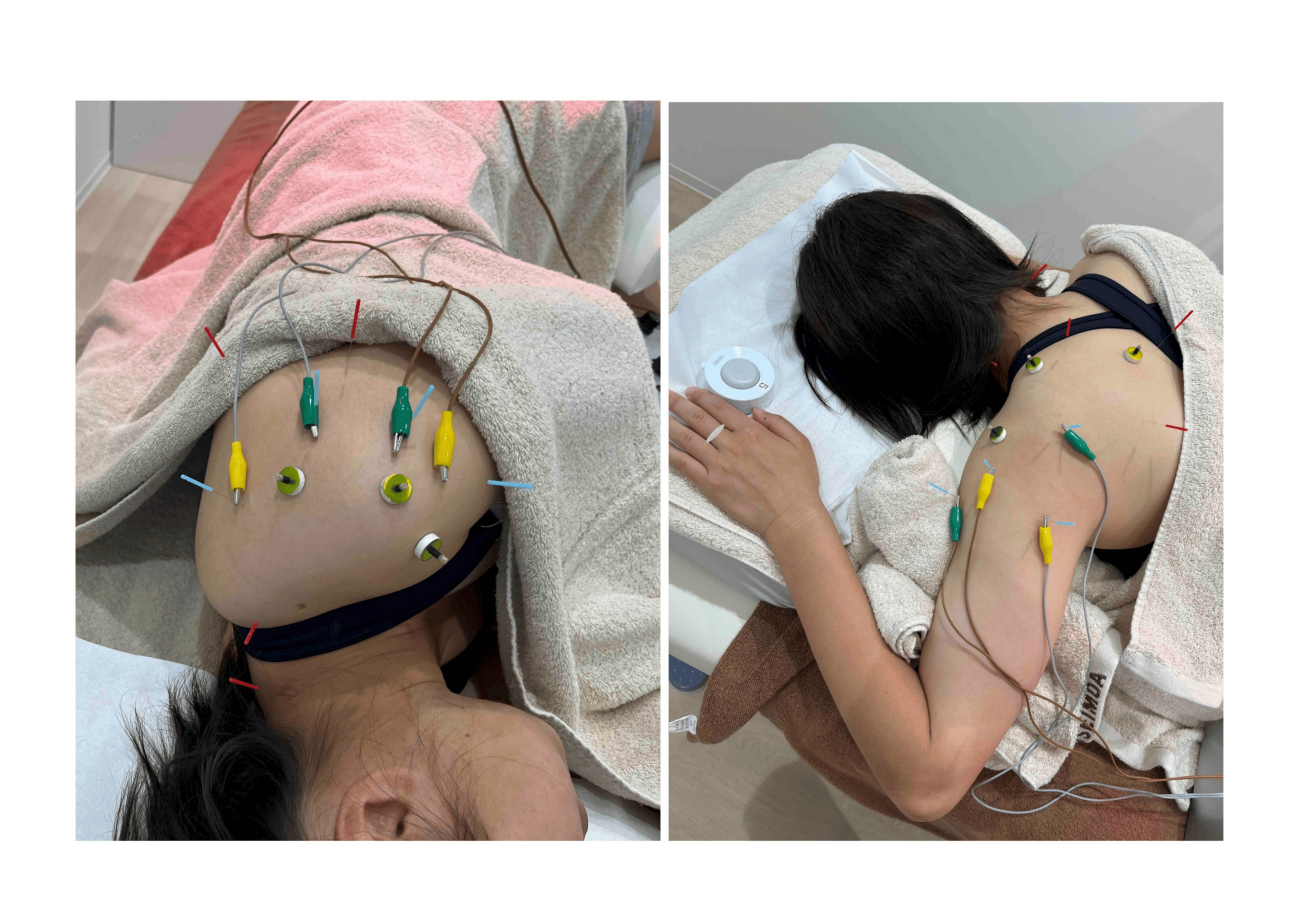
Acupuncture and moxibustion treatment for shoulder pain Left panel: Treatment targeting pain in the anterior and posterior regions of the left shoulder and lateral neck pain associated with a stiff neck. Right panel: Treatment targeting pain in the upper and posterior shoulder and the area near the medial border of the scapula.

Connective tissues that may cause pain as sequelae of RSD include the muscles surrounding the shoulder joint, the glenohumeral ligament, the coracohumeral ligament, the acromioclavicular ligament, the coracoclavicular ligament, the joint capsule, and the glenoid labrum. Considering the athlete's complaints of pain in the anterior shoulder, upper shoulder near the medial border of the scapula, posterior upper arm, and lateral neck due to sleeping awkwardly, acupuncture and moxibustion treatment were performed as shown in Figure 1. Disposable needles (Seirin Co., Shizuoka, JPN) were used for treatment. Electroacupuncture stimulation (needles: length 50 mm, diameter 0.20 mm; electrical current: frequency 5 Hz, amplitude 200 μs, intensity causing muscle contraction) was applied to the anterior and posterior deltoid regions where the patient perceived pain. Retaining needles (needles: length 20 mm, diameter 0.16 mm) were used at other reactive points. Needle insertion depth was approximately 1 cm (to reach the fascia). Moxibustions (Senefa Co., Shiga, Chikubushima, JPN) were also applied to enhance blood flow. Acupuncture treatment was administered seven times, with a frequency of once every seven to 10 days, with each session lasting 40 minutes. The total treatment period was one and a half months. The patient was informed about the acupuncture methods, and informed consent was obtained for data publication. This study was also approved by the Ethics Review Committee of Niigata University of Health and Welfare (approval no. 19294-240604).

**Figure 2:**
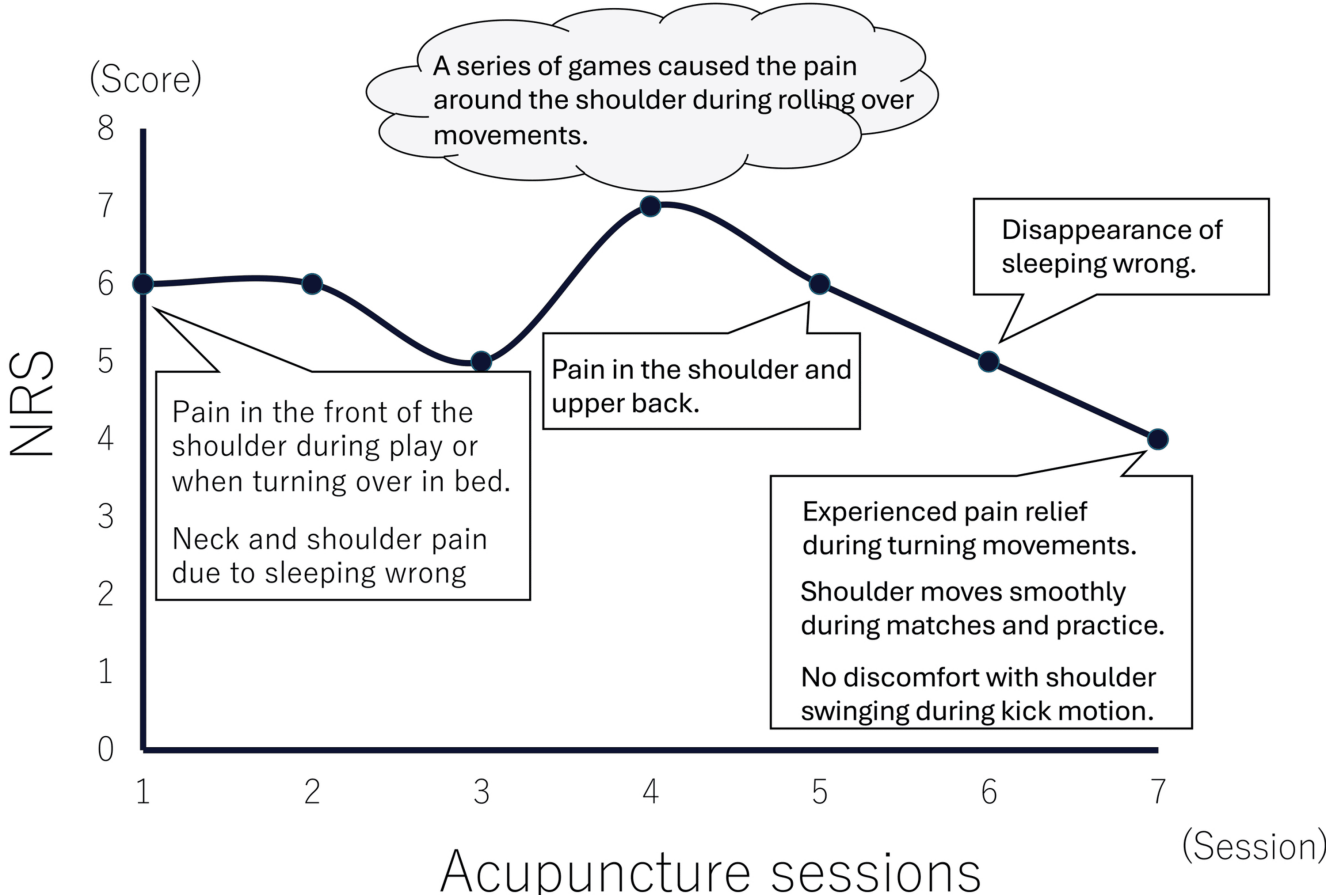
Changes in NRS following acupuncture and moxibustion treatment The vertical axis represents NRS, and the horizontal axis represents the acupuncture session. The NRS score was 6 at the first session. After a series of games, it was 7 (fourth session), but decreased to 4 at the seventh session. NRS: Numerical rating scale This diagram was created by the authors using Microsoft PowerPoint (Microsoft Corp., Redmond, WA, USA).

The athlete was instructed to continue inner muscle training for the rotator cuff using resistance bands and to continue rehabilitation with a physical therapist. The Numerical Rating Scale (NRS) score was 6 at the first session, and increased to 7 at the fourth session due to consecutive games. It decreased to 4 at the seventh session (Figure 2).

**Figure 3:**
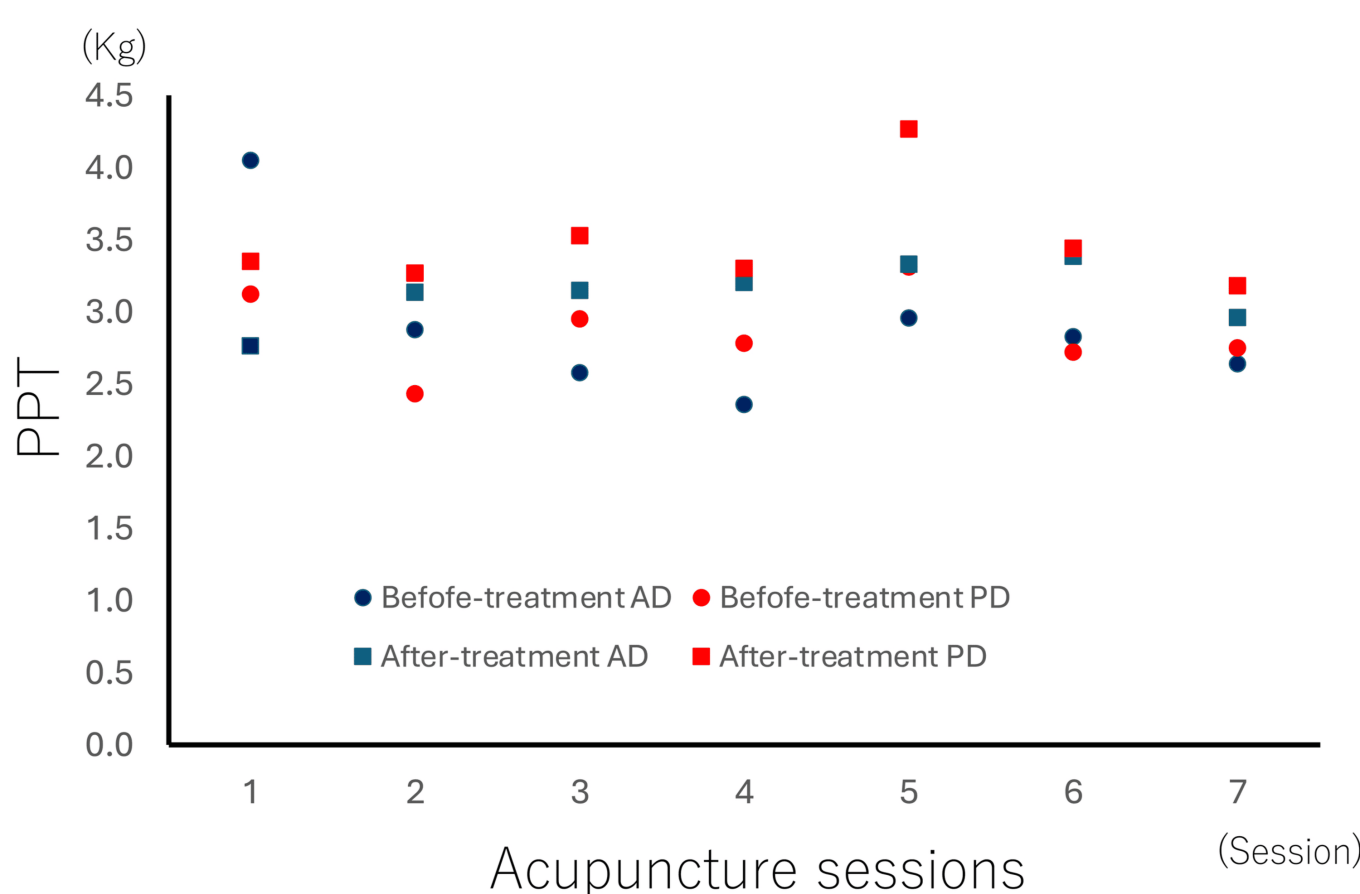
Changes in PPT at the site of pain following acupuncture treatment sessions The vertical axis represents PPT, and the measurement unit for PPT is expressed in kilograms applied during compression. The horizontal axis represents the acupuncture sessions. The PPT was measured in both the anterior deltoid (AD) and the posterior deltoid (PD). Blue circles indicate the PPT of the AD before treatment in each session. Blue squares indicate the PPT of the AD after treatment in each session. Red circles indicate the PPT of the PD before treatment in each session. Red squares indicate the PPT of the PD after treatment in each session. The interval between sessions was seven to 10 days. AD: Anterior deltoid, PD: Posterior deltoid, PPT: Pressure pain threshold This graph was created by the authors using Microsoft Excel (Microsoft Corp., Redmond, WA, USA).

Next, changes in pain threshold before and after each treatment session were evaluated. Pain threshold is an easily applicable indicator for objectively quantifying pain in clinical settings and sports environments. For pain threshold measurement, a device applied progressive pressure to the athletes' skin, recording the force (kg) at which pain was perceived. The measurement sites were the anterior and posterior fibers of the deltoid muscle, where the athletes reported pain. The pressure pain threshold (PPT) increased after each session, with an overall average change of +0.4 kg (approximately +14%). Notably, the change in the anterior deltoid after four sessions was +0.8 kg, and the change in the posterior deltoid after five sessions was +0.9 kg, indicating larger changes. This may correlate with the higher NRS scores (fourth session) and increased pain intensity observed during consecutive matches. Considering the single-case nature of the data, results are presented descriptively without inferential statistics (Figure 3). After the seventh treatment session, the athlete reported decreased shoulder heaviness, smoother shoulder movement, less noticeable pain during shoulder elevation during kicking motions, and smoother neck movement.

## Discussion

Recurrent shoulder dislocation is common in young individuals, and rehabilitation is highly important, as improving muscle function is necessary to correct joint instability. After the initial dislocation, if damaged periarticular tissues fail to repair properly, even minor external forces can cause redislocation in the same direction. The condition characterized by two or more dislocations is termed RSD. Shoulder dislocations are classified as anterior or posterior based on the position of the dislocated humeral head, with anterior dislocations accounting for more than 95% of cases and posterior dislocations representing 2% to 4% [1,2].

The female soccer player in the present case had a posterior shoulder dislocation, which is less common. The mechanism of injury typically occurs when axial compression is applied to the humerus while the shoulder is in an adducted and internally rotated position. Reports indicate that diagnosis can be difficult with plain radiographs alone, leading to frequent misdiagnosis [9]. For posterior shoulder dislocations, manual reduction is the first-line treatment when the humeral head defect is small or when the injury occurred within three weeks. Surgical reduction is indicated for delayed or chronic cases or when the defect is large, making manual reduction difficult [10]. Cicak reported that manual reduction for posterior shoulder dislocation is achieved by elevating and internally rotating the upper limb while applying traction to push the humeral head back into place [10]. However, reduction techniques vary widely, with representative methods including the Hippocrates, Milch, Kocher, and Stimson techniques [11-14].

In all cases, the fundamental principle during reduction is to perform the maneuver gently to reduce tension in the muscles surrounding the shoulder joint and avoid secondary injury. The immobilization period after manual reduction is typically three to four weeks using a sling, splint, or bust band, provided the reduced joint is stable. Regarding the immobilization position, fixation in shoulder external rotation reportedly yields favorable prognostic outcomes. This position helps approximate tissues when separation occurs at the injury site, leading to reduced dislocation anxiety, lower recurrence rates, and higher rates of return to sports [15-17]. In this case, reduction was performed by a physician at age 14 and self-reduction at age 17. The athlete does not recall which reduction technique was used, making identification difficult. However, reduction was confirmed to have been performed without anesthesia. Considering the patient's statement that the shoulder was immobilized in external rotation with a sling for three weeks, the reduction was likely performed with relaxed shoulder muscles using a conservative approach.

Rehabilitation for RSD requires strengthening the muscles surrounding the shoulder joint. Specifically, balanced strengthening of the inner muscles, namely the subscapularis, infraspinatus, and teres minor, to enhance shoulder internal and external rotation, contributes to shoulder stabilization. Strengthening the outer muscles, such as the deltoid and pectoralis major, is also crucial for preventing the recurrence of dislocation. Furthermore, sports involving overhead movements require shoulder joint flexibility [18]. In soccer, the kicking motion requires the contralateral shoulder to be in an abducted, extended, and externally rotated position, necessitating flexibility with 'bend' or 'give.' Thus, achieving shoulder stability requires balanced development of both strength and flexibility. In this case, the sports trainer actively performed range-of-motion exercises to enhance shoulder flexibility as part of rehabilitation. Additionally, following the trainer's instructions, the athlete performed rubber-tubing exercises to train the shoulder girdle muscles and also performed push-ups. However, the athlete experienced pain in the anterior shoulder, upper scapular region, near the medial border of the scapula, and posterior upper arm during both soccer play and daily activities. Pain also occurred around the shoulder when turning over in bed. Therefore, acupuncture treatment was added to the existing rehabilitation regimen to alleviate pain and promote blood circulation.

Numerous reports describe the analgesic effects of acupuncture. For example, Niruthisard et al. [8] reported that the mechanisms of acupuncture analgesia include local physiological responses at the insertion site, inhibition of nociceptive signals at the spinal and central levels, and release of endogenous opioids and other biochemical mediators from peripheral and central sources. They also reported that acupuncture analgesia may target not only pain management but also the underlying causes of pain, such as inflammation, via autonomic pathways, as it modulates specific brain networks essential for sensory, emotional, and cognitive processing. In contrast, literature on the efficacy of acupuncture for shoulder dislocation is limited, although two relevant studies were identified. Ackerman et al. administered acupuncture as an alternative to analgesic prescriptions during the reduction of anterior shoulder dislocations. They reported analgesic effects and eliminated the need for procedural monitoring or prolonged observation by nurses [19]. Additionally, Zhu et al. [20] administered acupuncture treatment (10 sessions over five months) in addition to Western medical treatment to patients who re-injured their shoulders two months after arthroscopic surgery for anterior shoulder dislocation. The results showed reduced shoulder pain, resolution of range-of-motion limitations, and normal findings on MRI. Although this case involved the rare posterior shoulder dislocation, the significant increase in PPT and decrease in NRS following acupuncture treatment suggest effects similar to those reported by Zhu et al. [20]. Furthermore, a meta-analysis of acupuncture for shoulder pain management reported that acupuncture may reduce pain associated with impingement syndrome and rotator cuff disorders and may improve shoulder range of motion [6,7]. Based on these findings, the treatment targeting the rotator cuff muscles and the deltoid in this case may have contributed to pain modulation. This effect may have facilitated relaxation of the scapular stabilizing muscles and dynamic stabilizers rather than directly treating the dislocation itself, thereby potentially reducing discomfort during movement. Therefore, acupuncture may represent a useful adjunctive approach to support ongoing rehabilitation for athletes experiencing shoulder pain as a sequela of RSD. However, this case study has limitations. Since acupuncture was administered concurrently with rehabilitation and muscle-strengthening training, the effect of acupuncture alone cannot be determined. Although pain decreased during the period of acupuncture treatment, the single-case report prevents confirmation of a causal relationship.

Although RSD pain is localized to the lesion site, periarthritis of the shoulder or rotator cuff tendinitis may cause referred pain across the entire shoulder region and upper limb [21]. In this case, pain occurred not only in the lateral and anterior shoulder but also, on some days, from the medial border of the scapula to the upper shoulder and the posterior aspect of the upper arm. These findings suggest the need to consider treatment approaches targeting referred pain. Furthermore, previous reports indicate that addressing the coracobrachialis muscle can improve shoulder range-of-motion limitations [22]. To enhance therapeutic effects, acupuncture treatment should be performed while also considering approaches targeting referred pain and deep muscles. In this case, only PPT and NRS were used to assess RSD pain. Patient-reported outcome measures (PROMs) such as the Oxford Shoulder Instability Score and the Simple Shoulder Test are important tools for evaluating treatment outcomes in patients with shoulder instability [23]. Therefore, the use of PROMs might have yielded more reliable results.

## Conclusions

In an athlete with shoulder pain due to RSD sequelae, acupuncture combined with rehabilitation resulted in increased PPT values and decreased NRS scores after treatment. Daily shoulder fatigue was also reduced, and shoulder pain during kicking motions in competition was alleviated. While this single case cannot establish a causal relationship regarding whether acupuncture alone produced the analgesic effect, these findings suggest that acupuncture may benefit pain management in shoulder pain associated with RSD sequelae. Acupuncture and rehabilitation complement each other, and their combined use may contribute to overall athletic conditioning.
